# SOD3 overexpression alleviates cerebral ischemia‐reperfusion injury in rats

**DOI:** 10.1002/mgg3.831

**Published:** 2019-08-28

**Authors:** Shuaiqi Sun, Ning Gao, Xiqi Hu, Han Luo, Jun Peng, Ying Xia

**Affiliations:** ^1^ Department of Neurosurgery Haikou People’s Hospital, Xiangya Medical College Affiliated Haikou Hospital, Central South University Haikou China; ^2^ Department of Neurosurgery Yiyang Central Hospital Hunan China

**Keywords:** ischemic stroke, mesenchymal stem cells, protection, *SOD3*

## Abstract

**Background:**

Ischemic stroke is a deadly disease that poses a serious threat to human life. Superoxide dismutase 3 (SOD3, ECSOD) is the main antioxidant enzyme that removes superoxide anions from cells. This study aimed to investigate the effect of SOD3 overexpression on cerebral ischemia‐reperfusion injury in rats.

**Methods:**

GV230‐EGFP‐ECSOD, the recombinant SOD3‐overexpressed vector, was constructed by genetic engineering technology, and mesenchymal stem cells (MSCs) were infected with lentiviral packaging. In animal experiment, cerebral ischemia‐reperfusion injury model rats were successfully established. ECSOD‐MSCs are the MSCs that successfully transfected with SOD3 overexpression vector. The animals were injected with ECSOD‐MSCs (ECSOD‐MSC group), normal MSCs (MSCs group), PBS (PBS group), and not do any processing (Model group) via the tail vein. Then MRI was used to detect the infarct volume of rats, modified Neurological Severity Scores (mNSS), and immunohistochemistry were used to evaluate the expression of neurological function and apoptosis‐related genes in rats.

**Results:**

Western blot analysis revealed that the *SOD3* was highly expressed in MSCs. Animal experiments showed that the transplantation of ECSOD‐MSCs significantly reduced the infarct volume of ischemic stroke rats (*p* < 0.05), significantly improved neurological function in rats (*p* < 0.05), and found proapoptotic gene, Bax, expression was significantly decreased (*p* < 0.05), the expression of anti‐apoptotic gene, Bcl‐2, was significantly increased (*p* < 0.05). The highly expressed SOD3 has no correction with brain infarct volume, and the highly expressed SOD3 has a positive correlation with cell apoptosis. It is speculated that overexpression of *SOD3* affects the expression of Bax and Bcl‐2, and improves apoptosis to alleviate ischemic stroke.

**Conclusion:**

Our results indicated that MSCs transfected with *SOD3* can effectively alleviate cerebral ischemia‐reperfusion injury in rats.

## 1 INTRODUCTION

Ischemic stroke is a type of cerebrovascular disease (CVD) that threatens people's lives and is the leading cause of death worldwide (Malik et al., 2015; Paramasivam, 2015). Ischemic stroke accounts for about 80% of CVD, and it occurs mainly because of the cerebral blood artery embolism caused by atherosclerosis of cerebral vascular or thrombosis or vascular injury, which can cause cerebral ischemia and necrosis (Wiklund, Patnaik, Sharma, Miclescu, & Sharma, 2017). The key to treating ischemic stroke is to reconnect the occlusive vessels and restore the blood flow to the ischemic region, to save the brain tissue that faces the infarction (Kuo et al., 2017). At present, the pathogenesis of ischemic stroke is still unclear, and treatment methods are constrained.

The discovery of neural stem cells makes it possible to treat the neurodegeneration, cranial vascular disease, and central nervous system injury (Hardingham, Patani, Baxter, Wyllie, & Chandran, 2010; Jiang, Chen, Chen, & Shen, 2012). It is effective to transplant embryonic stem cells and neural stem cells in treating neurological diseases (Kwon, Ahn, & Kang, 2018); however, due to the inherent defects such as immunological rejection, difficulty in materials and ethics, the application is limited. In recent years, studies have shown that mesenchymal stem cells (MSCs) had the characteristics of stem cells, like highly self‐renewal and multi‐directional differentiation in vitro culture (Wei et al., 2009); what's more, there is no specific surface antigen on MSCs and the implantation reaction is weak, so MSCs have been considered to be an ideal target cells in gene therapy (Liu et al., 2010). Yoo et al. (2008) exposed that MSCs can improve the symptoms of cerebral hemorrhage in animal neurologic function with ischemia, it is shown that the MSCs can effectively promote the repair of nerve injury caused by cerebrovascular disease.

Ischemic stroke damages the nervous system through the oxygen free radical chain reaction, which aggravates brain tissue damage. Superoxide dismutase (SOD) is the main antioxidant enzyme that removes superoxide anions in cells. Liu et al. (2012) proved that leonurine could protect brain injury by promoting SOD activity. And extracellular SOD (SOD3 or ECSOD, HGNC Approved Gene Symbol: *SOD3*, 4p15.2; OMIM: 185490) is the most important SOD in extracellular humoral (O'Leary, Bellizzi, Domann, & Mezhir, 2013), such as lymphatic fluid, synovial fluid, and plasma. SOD3 is a weekly hydrophilic glycoprotein with a molecular weight of 135,000 approximately. Each subunit of SOD3 consists of a copper and a zinc atom, which are necessary for the enzyme to remain active (Belda et al., 2013). Recently gene therapy studies have indicated that SOD3 has a good effect on some diseases caused by superoxide free radical (Shuvaev et al., 2013), Hattan, Chilian, Park, and Rocic (2007) found that *SOD3* intervention can alleviate the symptom damage in coronary atherosclerosis.

Although the effect of MSCs on ischemic stroke in rats has been studied, the effect of MSCs cells transfected with *SOD3* on ischemic stroke has not been reported until now. Therefore, the purpose of this study was investigating the effect of *SOD3* transfection with MSCs on ischemic stroke.

## 2 MATERIALS AND METHODS

### 2.1 Experimental animal

The animal experiment program was approved by the Animal Ethics Committee of Haikou People's Hospital and Yiyang Central Hospital. Male Sprague Dawley (*SD*) rats were purchased from Tianqin Laboratory Animal Center (Changsha, China). All rats were caged in an approved animal facility with free access to food and water, and were kept in a temperature‐controlled environment in a 12 hr light/dark cycle. All animal experiments were conducted in accordance with the United States National Institutes of Health Guide for the Care and Use of Laboratory Animals (NIH Publication No. 85‐23, revised 1996).

### 2.2 Animal study

The rat model of cerebral ischemia reperfusion was established (Liu et al., 2011) and 80 male *SD* rats (100–140 days, 240–280 g) were divided into 6 hr group (*n* = 40) and 12 hr group (*n* = 40) according to the time of ischemia, then treated with different drugs. ECSOD‐MSCs are the MSCs that successfully transfected with SOD3 overexpression vector. The animals were injected with 1 × 10^5^/μl ECSOD‐MSCs (ECSOD‐MSC group, *n* = 10), 1 × 10^5^/μl normal MSCs (MSCs group, *n* = 10), PBS (PBS group, *n* = 10) and not do any processing (Model group, *n* = 10) via the tail vein.

### 2.3 MSCs cell culture and SOD3 transfection

Rats (28 days, 100–150 g) underwent abdominal anesthesia and cervical dislocation. MSCs were isolated from the femur and tibia for culture. The phenotype of MSCs was detected by flow cytometry. Next, total RNA of rat MSCs was extracted, and the primer sequence was sod3(6885‐1)‐P1 TCCGCTCGAGATMSCGGTGGCCTTCTTGTTCTGC, sod3(6885‐1)‐P2 ATGGGGTACCGTAGTGGTCTTGCACTCGCTCTCC. *SOD3* was obtained by double digestion, and GV230‐EGFP‐ECSOD, the recombinant SOD3‐overexpressed vector was constructed. The production and titration of lentiviruses are based on the manufacturer's protocol.

The effect of *SOD3* transfection was determined by fluorescence microscopy (×100) and real‐time quantitative PCR (qPCR). The experimental groups were as follows: vector infection group (con) without unlinked gene, group without vector infection (blank) and sod3 transfection (sod3).

### 2.4 Western blot

The cell culture medium was added to the RIPA lysate and centrifuged at 4 ℃ to obtain total protein. An equal volume of 5× loading buffer was added, mixed with boiling water for 5 min, the ice box was rapidly cooled, the sample was loaded and electrophoresed. The protein was then transferred to the NC membrane and Ponceau staining (Sigma‐aldrich trading co. LTD, Shanghai, China) was used to determine the efficiency of protein transfer. Hybridization was performed with the original antibody overnight at 4°C. After TBS‐T washing, it was incubated with HRP‐labeled secondary antibody for 60 min and washed with TBS‐T. Pierce ECL Western Blotting Substrate (Thermo, Shanghai, China) was incubated with the NC membrane for 3 min to produce a rinse solution for analysis.

### 2.5 Detection of the effect of SOD3 transfection on MSCs

Three groups of cells were digested by 0.25% trypsin, forming single‐cell suspension with a concentration of 1 × 10^5^/ml, plated them on 96‐well plate, and continuously cultured at incubator for 6 days at 37°C, 5% CO_2_. 150 μl DMSO measured the optical density (OD,) values at 490 nm every day, then the cell growth curve was plotted.

Three groups (sod3, con, and blank) of cells were added to the 96‐well plate, then by using EdU DNA Proliferation in vitro Detection kit (C10310, Guangzhou RiboBio Co., LTD, Guangdong, China) cell proliferation was detected according to the manufacturer's instructions. The staining was observed under a fluorescence microscope, and the image was obtained for EdU analysis.

### 2.6 Imaging for determination of infarct size in rats

Four groups (ECSOD‐MSCs, MSCs, PBS, and Model) of rats were scanned by Magnetic Resonance Imaging (MRI) (GE signa HDX 3.0T MRI) at 1 day and 28 day after surgery to evaluate the changes in the area and volume of cerebral infarction.

$$\begin{array}{c} \text{Relative infarct volume}\\ \;=(\text{Initial infarct volume}-\text{terminal infarct volume})/\text{initial}\\ \;\;\text{infarct volume}\;\times 100\% \end{array}$$

### 2.7 Evaluation of neurological function

Evaluating modified Neurological Severity Scores (mNSS) scores in different time points: 1 day before the surgery, and after ischemia reperfusion at days 1, 3, 7, 14, 28, respectively, the scores mainly include exercise, feeling, balance, and reflection. One point is defined as failure to complete an experiment or test no response, and the higher the neurological deficit, the higher the score. The details were showed in Table S1.

### 2.8 Immunohistochemistry

Brain tissue sections were placed in 5 μm thick paraffin for immunohistochemical staining, heating 0.01 M citrate buffer (pH = 6.0), in which tissue sections were soaked to repair antigen, and sections were incubated in 3% H_2_O_2_ to extinguish. And then, the endogenous enzyme was incubated, 50–100 μl of anti‐rat/rabbit HRP‐labeled polymer was added to incubate IgG‐HRP, and background stained sections were washed with PBS. Dyeing was performed using DAB operating fluid; sections were counterstained with hematoxylin, then dehydrated with alcohol and fixed with neutral glue, and finally observed under a microscope.

### 2.9 Statistical analyses

The experimental data were collated using Excel 2016 and analyzed using SPSS 21.0. Differences between groups were compared by one‐way analysis of variance, and differences between 6 hr and 12 hr were assessed by *t* test. The software of Quantity One analyzed the peak gray value of the western blot. Images were collected for immunohistochemical data analysis according to software Image‐pro‐plus (IPP). The results were described as mean ± standard deviation, *p* < 0.05 indicates that the difference was statistically significant.

## 3 RESULTS

### 3.1 Successful acquisition of MSCs cells

The morphological changes of MSCs during the culture process were shown in Figure S1. Flow cytometry analysis results were showed in Figure S2. The positive expression rates were as follows: CD29 (β1 integrin) positive rate was 95.7%, CD90 (MSCs surface glycophosphatidyl inositol) positive rate was 99.4%, and negative expression was CD34. (The negative rate of hematopoietic stem cell and endothelial progenitor cells was 98.0%, and the negative rate of CD45 (pan‐white cell marker) was 99.6%).

### 3.2 Overexpression of the SOD3 in MSCs

**Figure 1:**
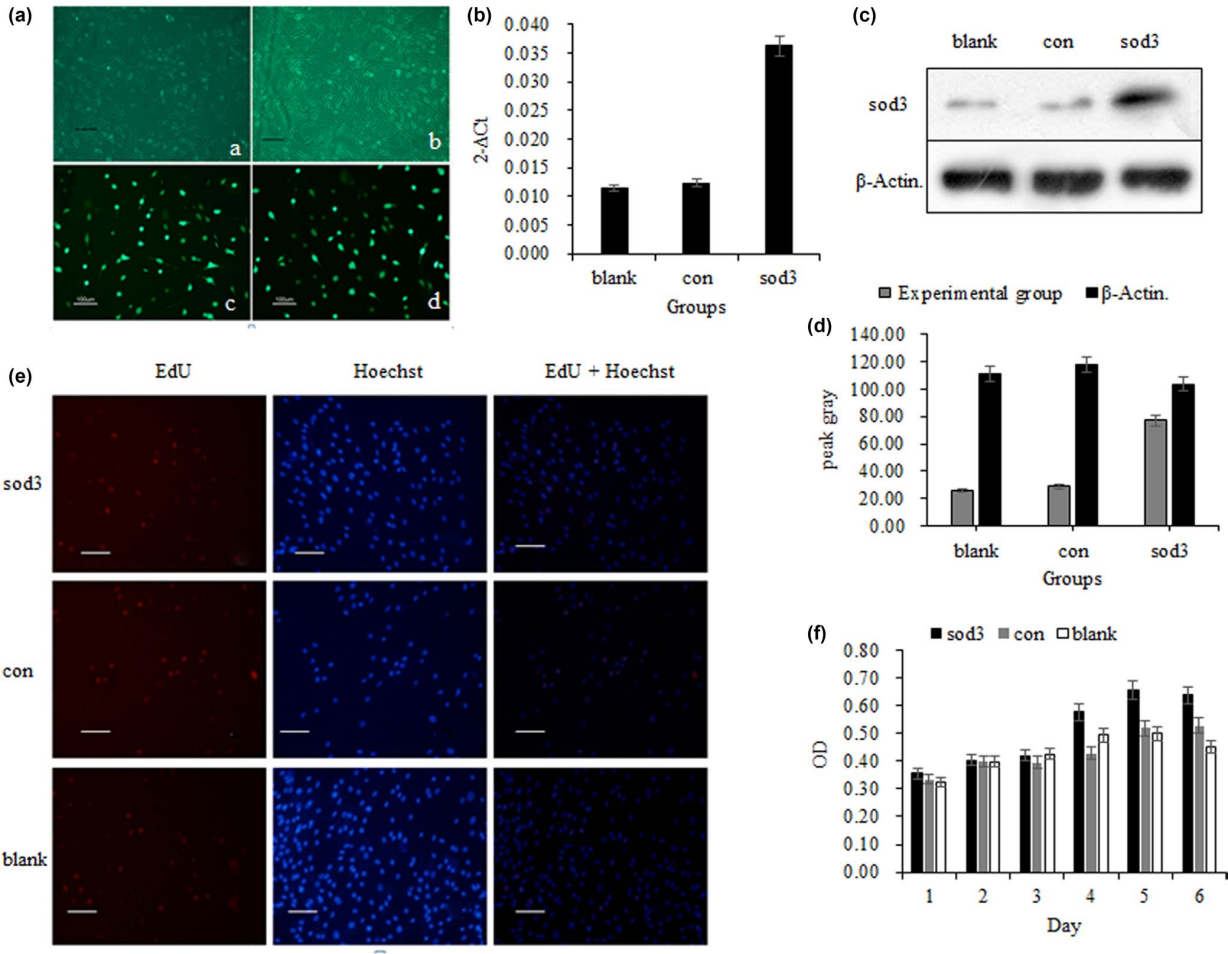
Effect of the *SOD3* on MSCs. A indicates the cell morphology observed by microscope (a is transfection 3d and b is 5d) and fluorescence microscope (c, d transfection 48 hr). (b) shows the content of mRNA carrying *SOD3* in MSCs cells analyzed by qPCR. (c) indicates the electrophoretic stripe results. (d) indicates the grayscale value of protein imprinting. (e) indicates the results of the EdU assay for detecting cell proliferation and viability. (f) represents the MSCs proliferation capacity. Blank indicates the proliferation of MSCs without transfected *SOD3*, con indicates the proliferation of MSCs with transfected empty vector, and sod3 indicates the proliferation of MSCs after transfected *SOD3*

Fluorescence microscopy successfully detected green fluorescent cells (Figure 1a), and the *SOD3* transfection efficiency was (75 ± 2.8)%. qPCR analysis showed that the mRNA expression level of the sod3 group was significantly increased (*p* < 0.05). The relative expression of the *SOD3* in the sod3 group was approximately three times that of the blank group and the control group (Figure 1b). In addition, Western blot results showed that the SOD3 expression in the sod3 group was significantly higher than that in the blank group and the control group (*p* < 0.05, Figure 1c and d).

### 3.3 Effect of SOD3 on proliferation of MSCs

MTT assay showed that *SOD3* transfection had no significant effect on cell proliferation of MSCs (*p* > 0.05, Figure 1f), that is, cell viability of MSCs was not affected after transfection of *SOD3*. And the EdU analysis showed similar results (*p* > 0.05, Figure 1e). It was suggested that the highly expressed SOD3 has no correction with cell proliferation.

### 3.4 ECSOD‐MSCs decreased brain infarct volume

**Figure 2:**
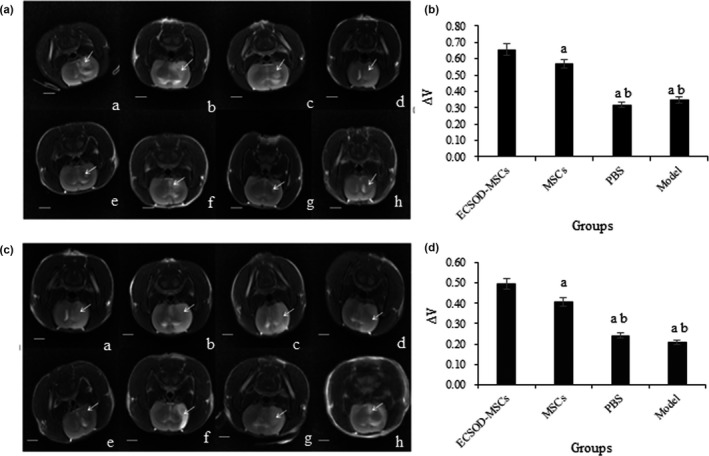
Shows the results of MRI examination after surgery in rats. (a, c) were MRI scan results of 6 hr and 12 hr groups respectively; a, b, c, d respectively indicated the infarct size of ECSOD‐MSCs group, MSCs group, Model group, and PBS group 1 day after operation; e, f, g, h respectively indicated the infarct size of the ECSOD‐MSCs group, the MSCs group, the Model group, and the PBS group at 28 days after operation. (b and d) were the results of the *t* tests in the 6 hr group and the 12 hr group, respectively. The arrow shows the infarction

The results of MRI examination in the rat brain infarction area were shown in Figure 2a and c. The infarct volume of the other three groups was statistically significant compared with the ECSOD‐MSCs group (*p* < 0.05): and compared with the MSCs group, the infarct volume was significantly changed (*p* < 0.05) in the PBS and Model groups (Figure 2b and d). It was suggested that the highly expressed SOD3 has no correction with brain infarct volume.

### 3.5 Administration of ECSOD‐MSCs improved neurological function

**Figure 3:**
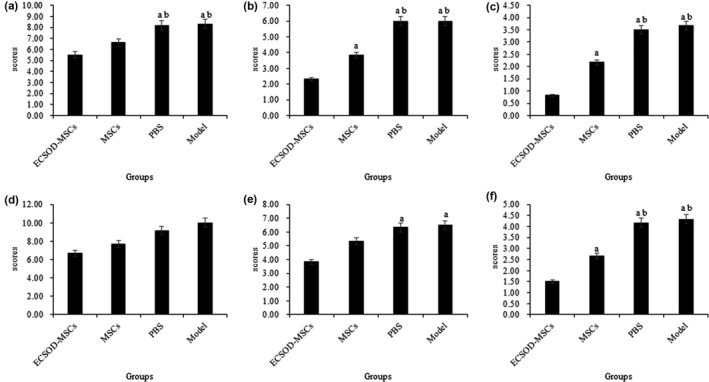
The mNSS scores at different time points after cerebral infarction in the 6 hr and 12 hr subgroups. The mNSS scores at 7 days (a), 14 days (b) and 28 days (c) in the 6 hr subgroups; The mNSS scores at 7 days (e), 14 days (f) and 28 days (g) in the 12 hr subgroups; a indicates that the group compared with the ECSOD‐MSCs group *p* < 0.05; b indicates that groups compared with MSCs *p* < 0.05

The mNSS scores at different time points after cerebral infarction in the 6 hr and 12 hr subgroups were presented in Figure 3 and Tables S2 and S3. The results showed that the mNSS scores at 14 day and 28 day after surgery at ECSOD‐MSCs groups were significant differences compared with three other groups (*p* < 0.05). The score of 7 day after surgery was not significantly different in the ischemic 12 hr group, but significantly different in the 6 hr group (*p* < 0.05). The mNSS scores were significantly different in PBS group and model group compared with the MSCs group (*p* < 0.05) at the time points of postoperative 7 day, 14 day, and 28 day.

### 3.6 ECSOD‐MSCs reduced the apoptosis

**Figure 4:**
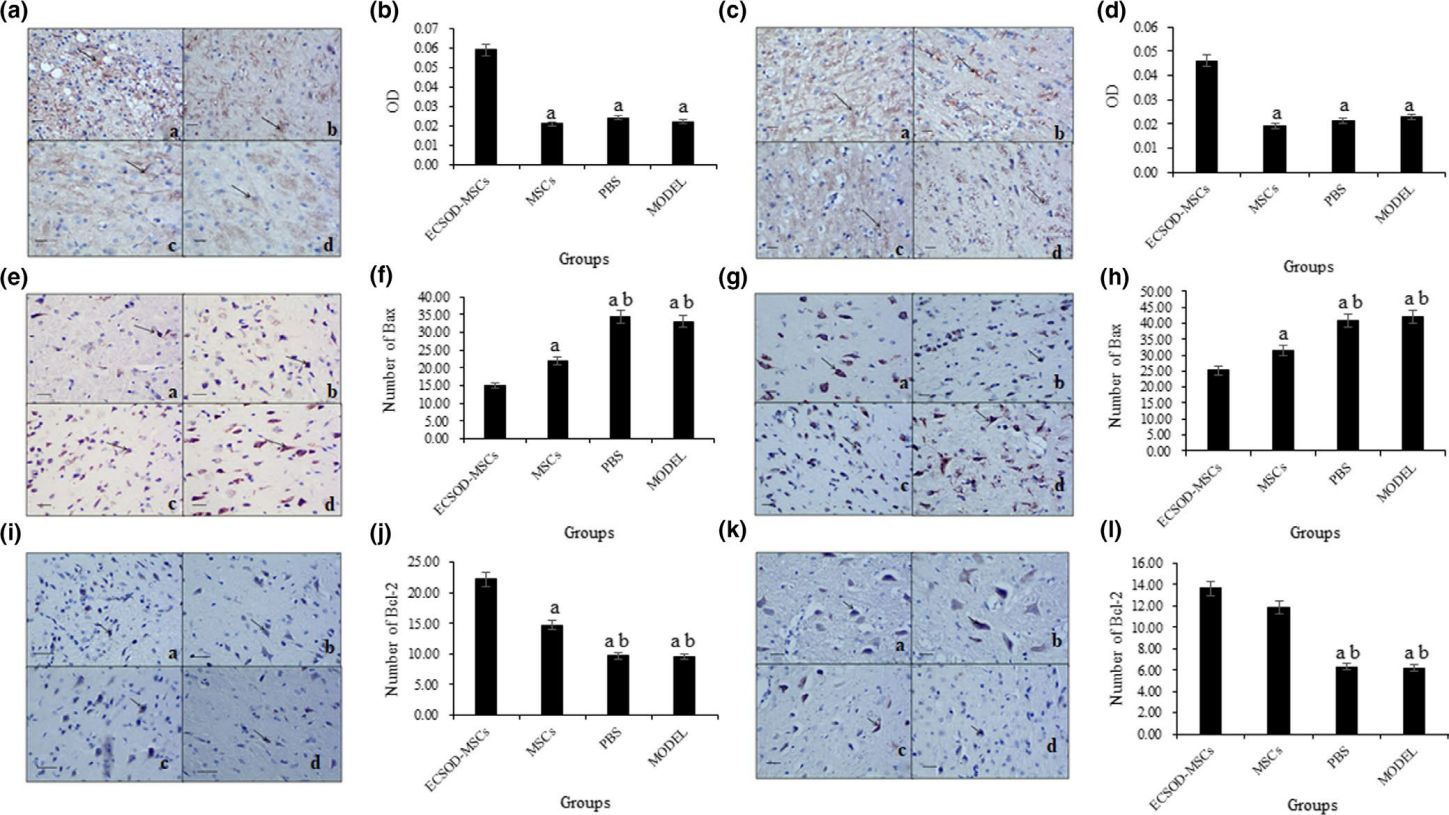
The results of immunohistochemical detection. (a) indicates that the group compared with the ECSOD‐MSCs group *p* < 0.05; (b) indicates that groups compared with MSCs *p* < 0.05. (a and b) represents the expression of *SOD3* in cytoplasm in 6 hr group. (c and d) are the expression of *SOD3* in cytoplasm in 12 hr group. (e and f) indicate the number of Bax‐positive cells in the 6 hr group. (g and h) are the number of Bax‐positive cells in the 12 hr group. (I and j) are the number of Bcl‐2–positive cells in the 6 hr group. (k and l) show the number of Bcl‐2–positive cells in the 12 hr group

Immunohistochemistry results showed that the expression of *SOD3* in ECSOD‐MSCs group was significantly higher than that in the other three groups (*p* < 0.05), but there was no statistical difference among the MSCs group, the Model group, and PBS group (Figure 4a–d).

The Bax‐positive cells in PBS and Model groups were significantly higher than that in ECSOD‐MSCs group and MSCs group (*p* < 0.05), What's more, the Bax positive cells in ECSOD‐MSCs group was significantly lower compared to the other three groups (*p* < 0.05, Figure 4e–h).

Furthermore, compared with the other three groups, the number of Bcl‐2–positive cells in the ECSOD‐MSCs group was significantly higher in 6 hr group (*p* < 0.05), and compared with PBS group and Model group, the Bcl‐2 cells in MSCs group were significantly increased (*p* < 0.05, Figure 4i,j). In 12 hr group, the Bcl‐2–positive cells in group ECSOD‐MSCs and MSCs were significantly higher than that in PBS group and Model group (*p* < 0.05, Figure 4k,l). It was suggested that the highly expressed SOD3 has a certain correlation with cell apoptosis.

## 4 DISCUSSION

Our study showed that *SOD3* was transfected into MSCs, which upregulated the expression of *SOD3* in cerebral ischemic tissue, leading to a reduction in infarct volume and ultimately improved neurological recovery in a rat stroke model.

Previous studies have shown that MSCs play an important role in stroke disorders. Abiko et al. (2018) suggested that cMSCs had potential as a candidate cell‐based therapy for stroke. Wu et al. (2018) indicated that intracerebral transplantation of Wharton's jelly‐derived mesenchymal stromal cells (WJ‐MSCs) reduced neurodegeneration and inflammation in the stroke brain. We found that MSCs transplantation can significantly reduce the infarct volume and restore neurological damage in cerebral ischemia rats, especially the MSCs transfected with *SOD3*. Lin et al. (2013) showed that MSCs transplantation improves functional recovery and reduces the inflammatory responses in rats with cerebral ischemia. This research supports our results.

Moreover, we found that MSCs transfected with the *SOD3* had a more pronounced effect on the relief of nerve damage. SOD is the main antioxidant in the body, it can inhibit the damage of active oxygen to the organism, and repair the damage caused by free radicals in time (Zhang, Zhou, & Zhang, 2018). Shuvaev et al. (2013) found that endotoxin‐induced cerebral vascular leukocyte adhesion was reduced by injecting Ab/SOD into mice, demonstrating that *SOD* could protect the brain from ischemia‐reperfusion injury. Förster and Reiser (2016) proved that *SOD3* can protect brain from peroxide damage in rats. It indicates that *SOD3* has protective effect on the damage caused by ischemia‐reperfusion injury. In our study, with the ischemic rat model of SOD3‐MSCs transplantation, the volume of cerebral infarction was significantly reduced, and the neurological function was significantly restored, indicating that the *SOD3* has therapeutic effects on ischemic stroke. Sun et al. (2016) believe that upregulation of SOD3 can alleviate the damage caused by cerebral ischemia. Liu et al. (2012) improved the neurological damage in ischemic stroke rats by enhancing the activity of the *SOD3*. Additionally, Jun, Fattman, Kim, Jones and Dory (2011) demonstrated that SOD3 plays an anti‐inflammatory role and inhibits the asbestos‐induced injury in 129/J strain of mice. These are consistent with our results, demonstrating that the *SOD3* is effective in ameliorating the damage caused by ischemic stroke.

The results of immunohistochemistry showed that the number of Bax‐positive cells was significantly decreased and the number of Bcl‐2 cells was significantly increased in rats transfected with ECSOD‐MSCs and MSCs. The Bax gene was considered to be a proapoptotic gene, while Bc1‐2 had the opposite biological function (Gawaly, 2016). It was suggested that ECSOD‐MSCs and MSCs had a mitigating effect on cerebral ischemic injury by altering the expression of Bax and Bcl‐2 genes. Miao et al. (2016) confirmed that Bcl‐2 expression was upregulated and Bax expression was weakened in the protective study of the nervous system in rats with ischemic stroke. Liu et al. (2017) protect neurons from ischemic stroke by regulating the expression ratio of Bcl‐2/Bax. A study showed that the expression of SOD3 in bone marrow mesenchymal stromal cells reduced ROS level and cell apoptosis both in vivo and in vitro in ischemia/reperfusion injury (Pan et al., 2014). Similar to this result, anti‐apoptosis gene Bcl‐2 MSCs were also promoted by SOD3 overexpression in the present study. Therefore, we hypothesized that the *SOD3* can decrease the expression of Bax protein, and increase the expression of Bcl‐2, thereby slowing the apoptosis of neurons, contributing to cell survival and protecting the nervous system.

Our results indicated that MSCs transfected with *SOD3* can survived in the infarct area and continuously express the *SOD3*, this will not only reduce the damage to the reperfusion injury, but also promote the recovery of damaged brain tissue, which can improve the curative effect of reperfusion after ischemia.

## 5 CONCLUSIONS

In this experiment, we preliminarily speculated on the role of *SOD3* transfection of MSCs in the treatment of ischemic stroke. However, the specific mechanism of action needs to be further studied.

## CONFLICT OF INTERESTS

All authors declare that they have no conflict of interests.

## AUTHORS' CONTRIBUTIONS

SS and XH conducted experimental operations, data analysis, and draft manuscripts. HL participated in the research design, assisted in experimental operations, and performed statistical analysis. JP conceived the study, participated in its design and coordination, and helped in drafting the manuscript. YX designed, coordinated, and supervised the study and critically reviewed and discussed the manuscript. All authors have read and approved the final version of the manuscript.
