# Supplementary figures and images for: SOD3 overexpression alleviates cerebral ischemia‐reperfusion injury in rats
Source: Mol Genet Genomic Med. 2019 Aug 28;7(10):e00831. doi: 10.1002/mgg3.831 (PMC6785449; doi:10.1002/mgg3.831)

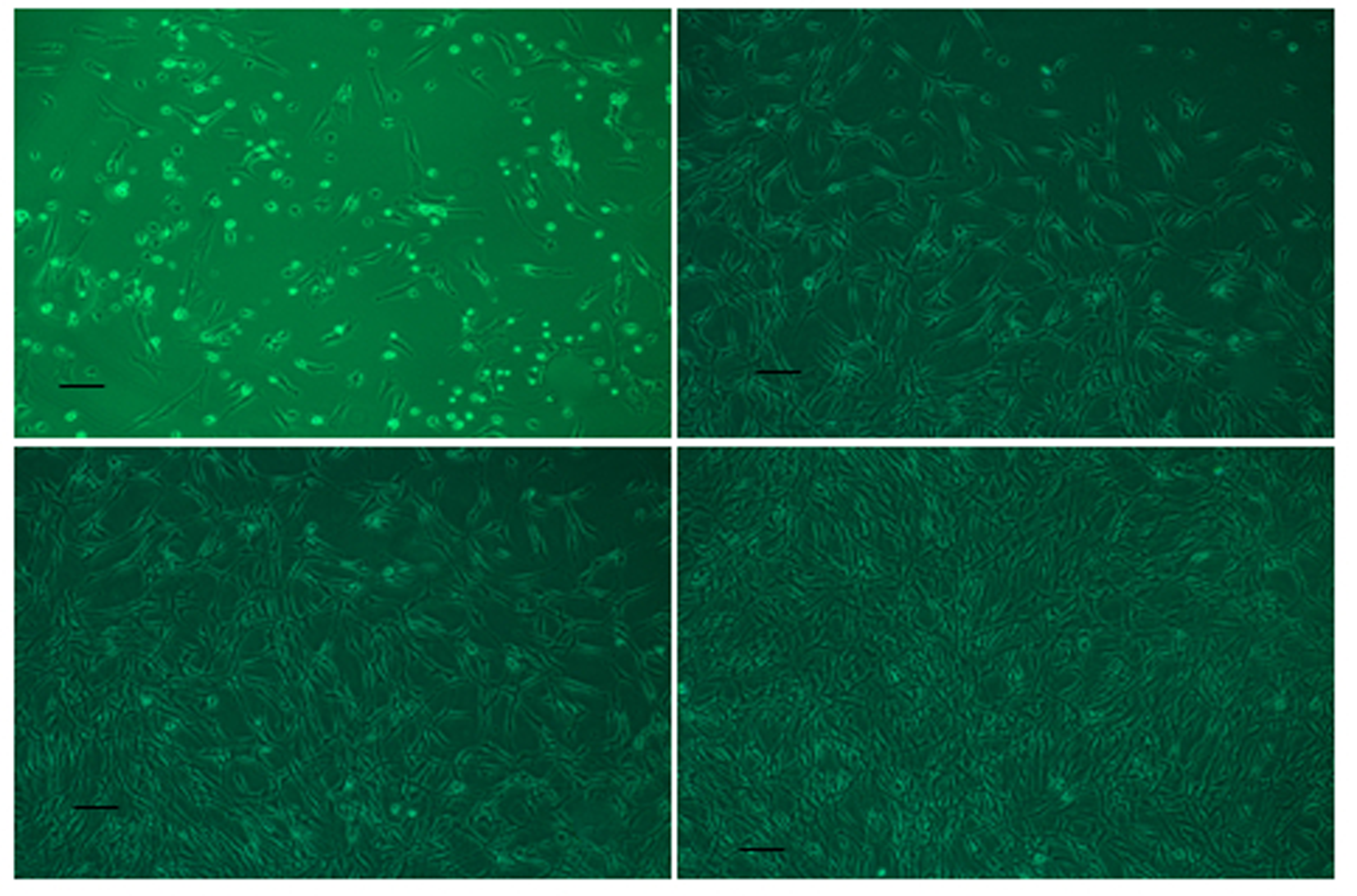

Supplement: Supplementary file 1 [file MGG3-7-e00831-s001.tif]

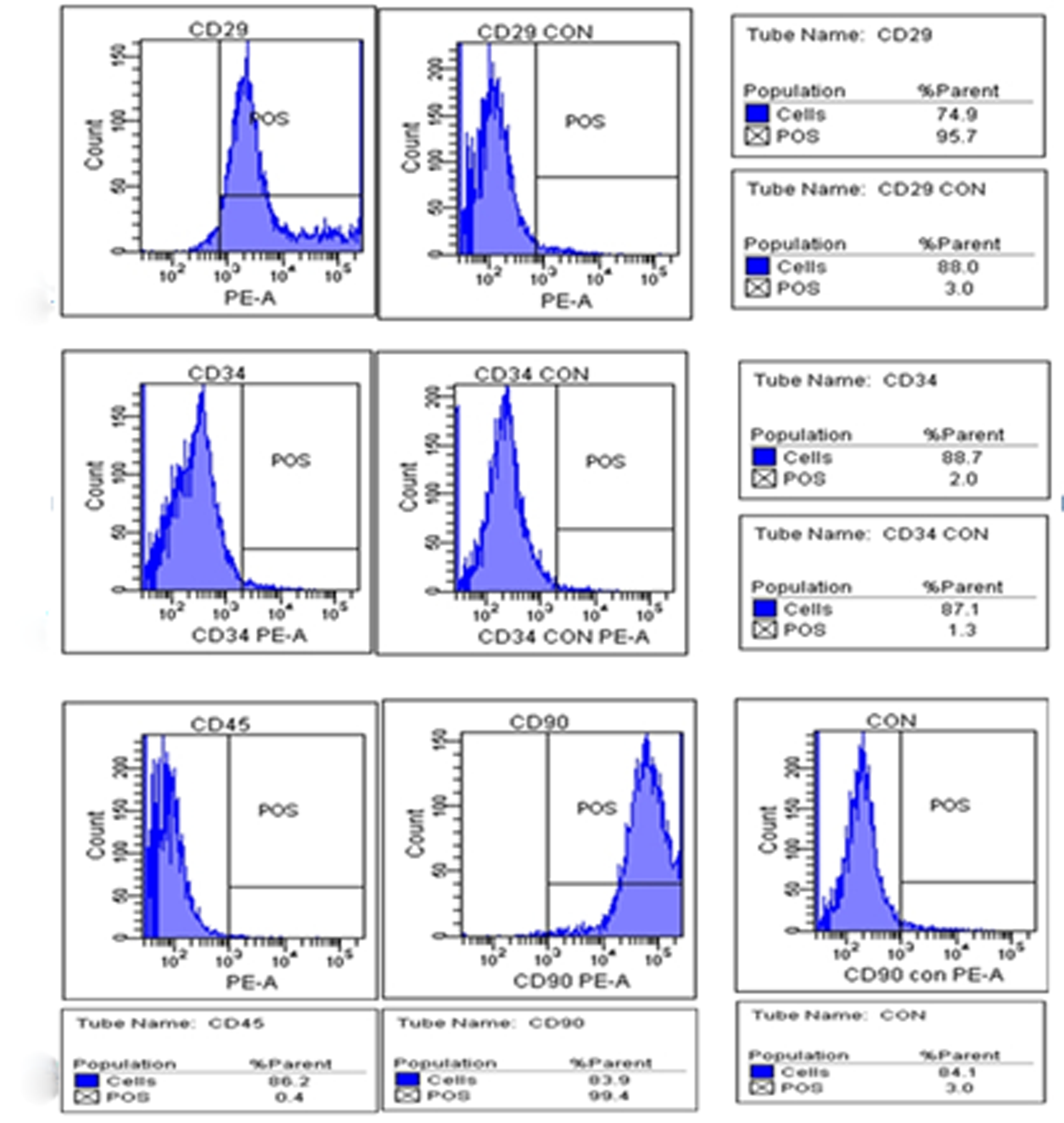

Supplement: Supplementary file 2 [file MGG3-7-e00831-s002.tif]
