# Supplementary material for: SOD3 overexpression alleviates cerebral ischemia‐reperfusion injury in rats
Source: Mol Genet Genomic Med. 2019 Aug 28;7(10):e00831. doi: 10.1002/mgg3.831 (PMC6785449; doi:10.1002/mgg3.831)
Supplement: Supplementary file 3 [file MGG3-7-e00831-s003.docx]

**Supplementary table 1** Modified neurological severity scores

| **Functional test** | **Score** |
| --- | --- |
| Motion testing |  |
| Filed a rat tail |  |
| Fore buckling | 1 |
| Hind legs buckling | 1 |
| The head deviation from the vertical line >10° in 30 seconds | 1 |
| The rats were placed on the table |  |
| Walk normally | 0 |
| Unable to walk in line | 1 |
| Circle the hemiplegia | 2 |
| Tip to hemiplegia | 3 |
| Sensory testing |  |
| Shelf test（visual sense and tactile sense） |  |
| The animals can't move by pushing | 1 |
| Proprioceptive test（deep sensation） |  |
| the animal has no body contractions, when placed the limb on the edge of the table | 1 |
| Balance beam testing |  |
| Postural Balance | 0 |
| Tighten the balance beam | 1 |
| Hold the balance beam tightly and fall off one limb | 2 |
| Hold the balance beam and fall off two limbs, or the balance beam to rotate >60s | 3 |
| Balanced for 40s on the balance beam and fall off | 4 |
| Maintain a balanced on the balance beam for 20s | 5 |
| Fall or balance on the balance beam less than 20 s | 6 |
| The reflection disappears and the abnormal movement |  |
| Loss of auricle reflex (shake head after stimulation of the ear canal) | 1 |
| The corneal reflection (cotton stimulates the cornea blink) disappears | 1 |
| The startle reflex (movement of sound) is gone. | 1 |
| There is an abnormal tension in the myclonic muscle | 1 |
| Total | 18 |
