# Supplementary material for: SOD3 overexpression alleviates cerebral ischemia‐reperfusion injury in rats
Source: Mol Genet Genomic Med. 2019 Aug 28;7(10):e00831. doi: 10.1002/mgg3.831 (PMC6785449; doi:10.1002/mgg3.831)
Supplement: Supplementary file 4 [file MGG3-7-e00831-s004.docx]

**Supplementary table 2** The mNSS scores at different time points after cerebral infarction in the 6 hours subgroups

| group | Preoperative 1 day | 1 day after surgery | 3 days after surgery | 7 days after surgery | 2 weeks after surgery | 4 weeks after surgery |
| --- | --- | --- | --- | --- | --- | --- |
| ECSOD-MSCs | 0 | 11.50 ± 2.07 | 8.50 ± 1.51 | 5.50 ± 1.05 | 2.30 ± 1.03 | 0.83 ± 0.75 |
| MSCs | 0 | 11.80 ± 2.40 | 9.67 ± 1.86 | 6.60 ± 1.21 | 3.83 ± 0.98^a^ | 2.17 ± 0.75^a^ |
| PBS | 0 | 12.17 ± 2.32 | 9.80 ± 2.04 | 8.17 ± 2.14^a b^ | 6.00 ± 1.55^a b^ | 3.50 ± 0.83^ab^ |
| Model | 0 | 12.00 ± 1.79 | 10.50 ± 1.87 | 8.30 ± 1.21^a b^ | 6.00 ± 1.09^a b^ | 3.67 ± 1.03^a b^ |

^a^ indicates that the group compared with the ECSOD-MSCs group *p* < 0.05;

^b^ indicates that groups compared with MSCs *p* < 0.05.
