# Supplementary material for: SOD3 overexpression alleviates cerebral ischemia‐reperfusion injury in rats
Source: Mol Genet Genomic Med. 2019 Aug 28;7(10):e00831. doi: 10.1002/mgg3.831 (PMC6785449; doi:10.1002/mgg3.831)
Supplement: Supplementary file 5 [file MGG3-7-e00831-s005.docx]

**Supplementary table 3** The mNSS scores at different time points after cerebral infarction in the 12 hours subgroups

| group | Preoperative 1 day | 1 day after surgery | 3 days after surgery | 7 days after surgery | 2 weeks after surgery | 4 weeks after surgery |
| --- | --- | --- | --- | --- | --- | --- |
| ECSOD-MSCs | 0 | 12.50 ± 2.16 | 10.50 ± 2.07 | 6.67 ± 1.75 | 3.83 ± 1.47 | 1.50 ± 1.04 |
| MSCs | 0 | 12.00 ± 2.37 | 10.67 ± 2.34 | 7.67 ± 1.51 | 5.33 ± 1.37 | 2.67 ± 1.03^a^ |
| PBS | 0 | 12.30 ± 2.16 | 11.00 ± 2.00 | 9.17 ± 2.04 | 6.33 ± 1.37^a^ | 4.17 ± 1.17^a b^ |
| Model | 0 | 12.50±2.07 | 11.17±2.04 | 10.00±2.00 | 6.50±1.38^a^ | 4.33±1.50^a b^ |

^a^ indicates that the group compared with the ECSOD-MSCs group *p* < 0.05;

^b^ indicates that groups compared with MSCs *p* < 0.05.
